# Effect of TRPV2 cation channels on the proliferation, migration and invasion of 5637 bladder cancer cells

**DOI:** 10.3892/etm.2013.1301

**Published:** 2013-09-13

**Authors:** QUANLIANG LIU, XINGHUAN WANG

**Affiliations:** Department of Urology Surgery, Zhongnan Hospital of Wuhan University, Wuhan, Hubei 430071, P.R. China

**Keywords:** bladder carcinoma, transient receptor potential channels, migration, proliferation, matrix metalloproteinase 2

## Abstract

Transient receptor potential vanilloid 2 (TRPV2), a nonselective cation channel, has become an attractive target gene for tumor studies due to its wide range of physiological and pathological functions. However, its specific role in bladder cancer development and progression remains unclear. The aim of the present study was to investigate the effects of TRPV2 on the proliferation, migration and invasion of 5637 bladder cancer cells *in vitro*. Rat TRPV2 cDNA was transfected into 5637 bladder cancer cells and changes in the behavior of the cells were detected. It was observed that TRPV2 enhanced bladder cancer cell migration and invasion; however, it did not affect cell proliferation *in vitro*. TRPV2 activity, which may be mediated by direct matrix metalloproteinase 2 (MMP2) regulation, is important in bladder tumor development and progression. The results of this study suggest that TRPV2 channels are a potential therapeutic target for bladder carcinoma.

## Introduction

Bladder carcinoma is the most common malignancy of the urinary tract in China, while transitional cell carcinoma is the most commonly diagnosed urothelial tumor (1). The prognosis of patients with non-muscle invasive bladder cancer is good, with five-year survival rates of 82–100%; however, patients with metastatic urothelial cancer have a poorer prognosis, with two-year survival rates of only 5–10% (2). The tumor cells develop a high tolerance for intrinsic and extrinsic defense systems and therapeutic procedures. Furthermore, tumor cells may infiltrate into the adjacent tissues and metastasize to remote organs and tissues and cause bleeding, infection and dystrophy, in addition to disrupting important organ functions. Ultimately, tumor cells migrate and invade various organs, which leads to the mortality of the patient. At present, an effective therapy for metastatic urothelial cancer remains unavailable.

Temperature-sensitive transient receptor potential vanilloid (TRPV) channels are critical contributors to normal pain and temperature sensations. These channels are Ca^2+^-permeable and contribute to intracellular Ca^2+^ homeostasis. However, the regulatory mechanism and the role of the TRPV2 channel in carcinogenesis has not yet been elucidated. TRPV2, the second member of the TRPV superfamily, was initially known as vanilloid receptor-like protein 1 and shares 50% homology with TRPV1 (3). TRPV2 contains six transmembrane domains that consist of a putative pore-loop region, a cytoplasmic amino terminus with three ankyrin-repeat domains, and a cytoplasmic carboxy terminus. As a nonselective cation channel with high Ca^2+^ permeability, it also acts as a heat sensor, with a temperature threshold of 50–52°C (4) and may be activated by 2-aminoethoxydiphenyl borate (5) and insulin-like growth factor-1 (6). TRPV2 is widely distributed in human organs and tissues, such as the brain, vascular smooth muscle cells, the gastrointestinal tract, macrophages and the urothelial tract (7). Furthermore, TRPV2 has a wide range of physiological and pathological functions (8). Previous studies have shown that TPRV2 may be clinically associated with cancer (9–11), particularly urinary tract tumors (3,12,13). TRPV2 expression levels have been directly correlated with the tumor stage and grade of urothelial carcinoma (UC) of the human bladder (14). It has also been demonstrated that TRPV2 activation induces apoptotic cell death in human T24 bladder cancer cells (15). However, the role of TRPV2 in bladder cancer development and progression remains unclear.

The aim of this study was to investigate the effects of TRPV2 on the proliferation, migration and invasiveness of 5637 bladder cancer cells, which are characterized by low TRPV2 expression.

## Materials and methods

### Cell culture

Human 5637 bladder carcinoma cells were obtained from the American Type Culture Collection (Manassas, VA, USA) and cultured in RPMI-1640 medium (Gibco-BRL, Grand Island, NY, USA) supplemented with 100 IU ml^−1^ penicillin G sodium, 100 *μ*g ml^−1^ streptomycin sulfate and 10% fetal bovine serum (FBS; Gibco-BRL) in a humidified 95% air and 5% CO_2_ atmosphere at 37°C.

### Permanent transfection of 5637 cells with TRPV2 cDNA

The 5637 cells were plated on a six-well plate and transfected at ~85% confluence with the rat TRPV2 encoding vector, pcDNA3.1 (+), using Lipofectamine^®^ 2000 (Invitrogen Life Technologies, Carlsbad, CA, USA), in accordance with the manufacturer’s instructions. The stably transfected clones were selected using Geneticin^®^ G418 (Sigma, St. Louis, MO, USA) at 400 *μ*g ml^−1^. Seven clones were identified using reverse transcription-polymerase chain reaction (RT-PCR) and western blot analysis. The selected clones were subcloned and maintained under selection pressure for an additional week.

### RT-PCR

Total mRNA was isolated from cells using TRIzol reagent (Invitrogen Life Technologies), in accordance with the manufacturer’s instructions. Briefly, 2 *μ*g total RNA was reverse-transcribed with oligo-d(T) (Invitrogen Life Technologies) and ThermoScript™ reverse transcriptase (Invitrogen Life Technologies) in a final reaction volume of 20 *μ*l. Subsequently, 5% of the samples were amplified by PCR, using the primers listed in Table I. The primer sequences were designed using Primer Express Software (PE Biosystems, Foster City, CA, USA) and synthesized by Invitrogen (Shanghai, China). Two pairs of TRPV2 primers, which are absent in human TRPV2, were designed using the rat TRPV2 mRNA as a template to confirm whether the plasmid was successfully transfected and expressed at the mRNA level. Glyceraldehyde-3-phosphate dehydrogenase (GAPDH) was used for the quantification of the sample DNA amplification. The DNA amplification conditions included an initial denaturation step at 95°C for 5 min; 30 cycles at 95°C for 30 sec, 60°C for 30 sec, 72°C for 30 sec; and a final extension step at 72°C for 7 min.

### Western blot assay

The protein expression of TRPV2, matrix metalloproteinase 2 (MMP2), and GAPDH was assayed by western blot analysis. Equal quantities of the protein (30 *μ*g) were separated using 10% sodium dodecyl sulfate polyacrylamide gel electrophoresis and transferred onto enhanced chemiluminescence nitrocellulose membranes (Amersham Biosciences, Piscataway, NJ, USA). Following this, anti-TRPV2-specific antibodies (code: sc-30155; Santa Cruz Biotechnology, Inc., Santa Cruz, CA, USA) [1:250 (v/v) with non-fat milk], MMP2 antibodies (code: 4022, Cell Signaling Technology, Inc., Danvers, MA, USA) [1:400 (v/v) with non-fat milk], and anti-GAPDH-specific antibodies (code: sc-137179, Santa Cruz Biotechnology, Inc.) [1:500 (v/v) with non-fat milk] were used for the analysis. Western blot analysis was performed as previously described (16). Each experiment was repeated three times with similar results. One representative experiment is shown.

### Cell proliferation assay

A 3-(4,5-dimethylthiazol-2-yl)-2,5-diphenyltetrazolium bromide (MTT) colorimetric assay was used to measure the cell proliferation. Briefly, the cells were plated at the initial density of 500 per well in 96-well plates (Corning Life Sciences, Corning, NY, USA), and the medium was changed 24 h later (day 0). Thereafter, until day seven, the medium was changed daily. The MTT assay was performed in accordance with the manufacturer’s instructions (Sigma). The absorbance at 570 nm was quantified on a microplate spectrophotometer (ASYS-Hitech GmbH, Municipality of Eugendorf, Austria).

### Cell cycle assay

The cells (~5×10^5^ per well) were incubated until 85% confluence and digested with 0.25% trypsin (Gibco-BRL). The cells were subsequently harvested and fixed overnight with 70% ethanol in phosphate-buffered saline (PBS; added dropwise) at 4°C and then resuspended in PBS containing 40 *μ*g ml^−1^ propidium iodide, 0.1 mg ml^−1^ RNase, and 0.1% Triton X-100 in a dark room. Following incubation at 37°C for 30 min, the cells were analyzed using a flow cytometer (Becton-Dickinson, San Jose, CA, USA) equipped with an argon ion laser at a wavelength of 488 nm. The cell cycle stage was then determined and analyzed.

### Scratch motility assay

The cells were cultured for 24 h as confluent monolayers in complete medium and then wounded by moving them across the well with a standard 200 *μ*l pipette tip. The wounded monolayers were then washed twice to remove non-adherent cells. Wound closure was monitored for 24 h from initial wounding using an inverted phase contrast microscope (Leica, Wetzlar, Germany). Wound closure was monitored for 24 h, as this was shorter than the doubling time of the 5637 cells. The distance between borders was estimated using four different fields from each sample. Four equidistant points in each image were measured to obtain a better estimate of the true width of the wounded area. The migration rate was expressed as a percentage of the control (5637 cells, 0 h) and calculated as the proportion of the mean distance between the borderlines caused by scratching and the distance that remained cell-free following regrowth. Three independent series of experiments were performed in quadruplicate.

### Transwell assay

The cells were seeded on the top of 8.0-*μ*m pore Transwell cell culture inserts (Corning Life Sciences), which were paved with Matrigel glue (diluted 1:4 with serum-free RPMI-1640 medium; Millipore, Billerica, MA, USA) at a density of 50,000 cells per well (24-well plate) in serum-free culture medium containing 0.1% bovine serum albumin. Subsequent to culture, the cells were stimulated to migrate across the filters using 10% FBS as the chemoattractant in the assay chambers. Following 24 h of incubation at 37°C, the noninvading cells on the Transwell plates were scraped off with a cotton swab, whereas the cells that migrated through the filter pores to the lower surface of the inserts were fixed for 30 min with 4% paraformaldehyde in PBS and stained with 0.1% crystal violet for 20 min. The cells under each filter were counted on five random examination fields (magnification, ×200) using an inverted phase contrast microscope (Leica). The data are expressed as the mean of four wells ± standard error of the mean.

### Statistical analysis

SPSS statistical software for Windows version 17.0 (SPSS, Inc., Chicago, IL, USA) was used to conduct the statistical analysis. All data are presented as the mean ± standard error of the mean. Each experiment was repeated at least three times. ‘n’ indicates the number of the cells per experiment, whereas ‘N’ indicates the number of experiments performed. A Tukey-Kramer test was used for statistical comparisons of the means and differences and P<0.05 was considered to indicate a statistically significant difference.

## Results

### Detection of TRPV2 protein in 5637-TRPV2, 5637-vector, and 5637 cells

**Figure 1.**
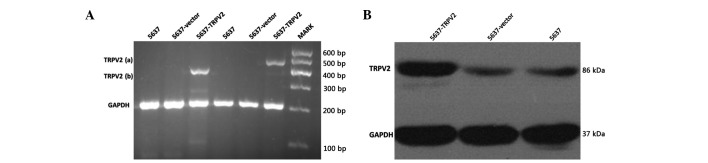
(A) Transient receptor potential vanilloid 2 (TRPV2) mRNA is expressed in the 5637-TRPV2 cells but not in the 5637 and 5637-vector cells. (B) Expression and intracellular distribution of TRPV2 protein in 5637-TRPV2, 5637-vector and 5637 cells. Glyceraldehyde 3-phosphate dehydrogenase (GAPDH) was used as the housekeeping gene. The results show significantly higher TRPV2 expression in the 5637-TRPV2 cells than in the other two cell types.

The two expected bands were detected in 5637-TRPV2 cells via an RT-PCR assay using specific primers (Fig. 1A). The result demonstrated that the plasmid was successfully transfected into the 5637 cells. The TRPV2 protein expression level was determined using western blot analysis (Fig. 1B). The TRPV2 protein expression levels in the 5637-TRPV2 cells were significantly higher than in the other cells, which indicated that the transfected plasmid was expressed at both the mRNA and protein levels.

### Effects of TRPV2 on 5637 cell proliferation

**Figure 2.**
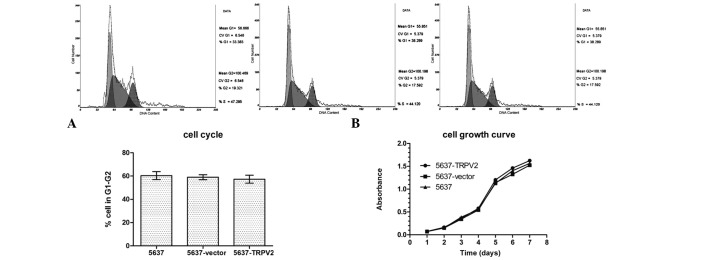
Effect of transient receptor potential vanilloid 2 (TRPV2) overexpression on 5637 cell proliferation. (A) Cell cycle distribution of the cells in the G1–G2 stage: 5637-TRPV2 (57.32±5.89%), 5637-vector (59.04±3.72%) and 5637 (60.36±5.89%). No significant differences were observed among the three cell groups (n=4, N=3; P>0.05). (B) No significant differences in the cell growth curves were observed among the three groups (n=8, N=3; P>0.05).

Cell proliferation was evaluated in terms of cell cycle distribution using flow cytometry. The percentage of cells in the G1–G2 stage was 57.32±5.89% for the 5637-TRPV2 group, 59.04±3.72% for the 5637-vector group, and 60.36±5.89% for the 5637 group. These results did not indicate any significant differences among the three cell groups (Fig. 2A). The results of the MTT assay also indicated a lack of significant differences among the cell growth curves of the three groups. These results suggested that the growth rate of the 5637 cells was unaffected by the TRPV2 channels (Fig. 2B) and that TRPV2 channels did not affect 5637 cell proliferation.

### Effects of TRPV2 on 5637 cell migration

**Figure 3:**
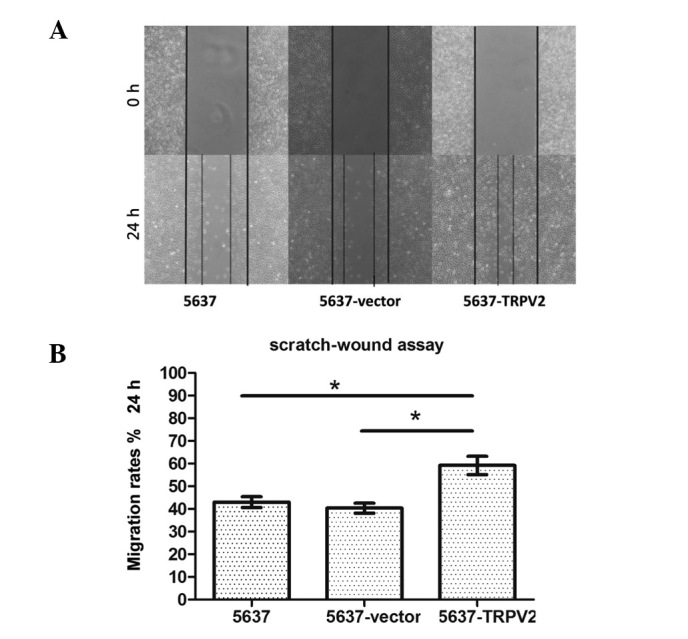
Transient receptor potential vanilloid 2 (TRPV2) enhances the migration and invasion of 5637 cells. (A) Representative views of the motility assays obtained at 0 h and 24 h (magnification, ×200). The broad dashed lines represent the original borders at the time of scratching, whereas the thin and solid lines represent the borders at 24 h subsequent to scratching. (B) Wound areas were measured and normalized relative to the control values (5637 at 0 h), which were assumed to be 100%. The data from three independent experiments were collected and their means were plotted with the standard error of the mean. The graph shows that the cell migration rates of the 5637-TRPV2 cells (59.21±4.04%) were significantly higher than the 5637 (42.99±2.37%) and 5637-vector (40.34±2.24%) cells (n=4, N=3; ^*^P<0.05).

A scratch-wound assay was used to observe the effects of TRPV2 on cell migration. Following 24 h of incubation, the motility of the 5637-TRPV2 cells was significantly higher than that of the cells in the 5637 and 5637-vector groups (Fig. 3A). Wound areas were measured and normalized relative to the control values (5637 at 0 h), which were assumed to be 100%. The results showed that the cell migration rate of the 5637-TRPV2 group (59.21±4.04%) was significantly enhanced compared with that of the 5637 (42.99±2.37%) and 5637-vector (40.34±2.24%) groups (n=4, N=3; P<0.05; Fig. 3B).

### Effects of TRPV2 on 5637 cell invasion

**Figure 4:**
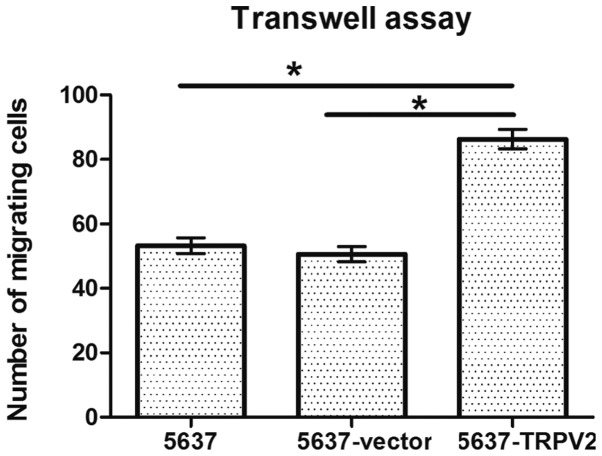
The cells under each filter were counted on five random examination fields (magnification, ×200). The data from three independent experiments were collected and their means were plotted with the standard error of the mean. The graph shows that the number of migrating 5637-transient receptor potential of vanilloid 2 (TRPV2) cells (86.31±7.04%) was significantly higher than that in the other groups: 5637 (53.22±4.94%) and 5637-vector (50.59±5.91%) cells (n=4, N=3; ^*^P<0.05).

A Transwell assay was used to observe cell penetration into the Matrigel glue. The number of migrating cells in the 5637-TRPV2 group (86.31±7.04) was significantly higher than that in the 5637 (53.22±4.94) and 5637-vector (50.59±5.91) groups (n=4, N=3; P<0.05; Fig. 4).

### Detection of MMP2 protein in 5637-TRPV2, 5637-vector and 5637 cells

**Figure 5.**
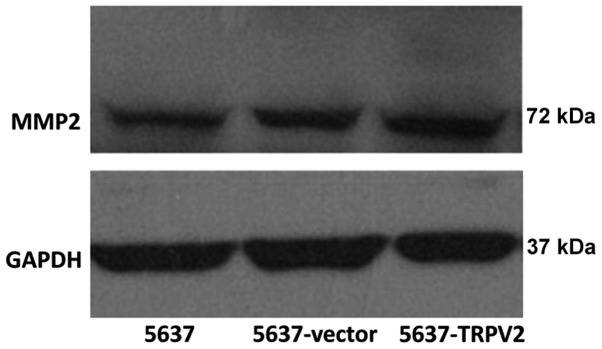
Changes in the protein expression of matrix metalloproteinase 2 (MMP2) and glyceraldehyde-3-phosphate dehydrogenase (GAPDH), shown by western blot analysis. The GAPDH protein expression in the same samples was used as the loading control. The results show that the protein expression level of MMP2 was significantly higher in the 5637-transient receptor potential of vanilloid 2 (TRPV2) cells than in the cells of the other two groups.

MMP2, a protein closely correlated with tumor cell migration, was detected using a western blot assay. MMP2 expression was higher in the 5637-TRPV2 group than in the other two groups (Fig. 5).

## Discussion

The TRPV family is a subclass of the TRP channel family. It contains six types of calcium-permeable channels that have unique channel properties (4,17,18). The TPRV family is expressed in a number of different tissues and functions to regulate cellular calcium metabolism and calcium signaling. Members of the TPRV family may also participate in cell proliferation, differentiation and apoptosis. As one of the important members of the TPRV superfamily, TRPV2 is highly expressed in sensory dorsal root ganglion neurons and the hypothalamus (7). It has been shown that TRPV2 is closely associated with the urinary system. For instance, TRPV2 is localized in the superficial cells, whereas there is negligible or poor expression in the basal and club-shaped cells in the urothelial cells. TRPV2 channels are expressed in normal human urothelial cells and in UC of the bladder. However, the expression level of TRPV2 channels in UC is significantly higher than in normal urothelial cells and is positively correlated with the clinical grade and stage of the tumor (14). It has been indicated that TRPV2 channels may be critical in the development and progression of bladder cancer, but their specific role remains unknown.

Yamada *et al* (15) studied functional TRPV2 expression in UC T24 and RT4 cells. Caprodossi *et al* (14) observed that the TRPV2 expression levels in UC cells were correlated with high-grade disease. However, unlike the present study, they focused on the regulation of calcium influx through TRPV2 channels, which induces apoptotic cell death in T24 cells. Nevertheless, they did not describe the changes in the proliferation and migration of the T24 cells under specific siRNA treatment. In the present study, the rat-TRPV2-encoding vector was transfected into 5637 cells, which are characterized by low levels of TRPV2 expression and relatively weak cell aggression, to verify the precise role of TRPV2 channels on bladder cancer (Fig. 1B). Following the selection of the monoclonal cell lines, no significant difference was observed in cell cycle distribution using flow cytometric analysis (Fig. 2A). Similar results were obtained from the cell growth curve in a week (Fig. 2B). These results indicated that TRPV2 exerted no effect on the cell growth rate and proliferation. The study also evaluated cell migration ability, with the results of the scratch-wound assay and the Transwell assay showing that TRPV2 enhanced 5637 cell migration.

TRPV2 channels contribute to intracellular Ca^2+^ homeostasis. Activation of the channels increases the intracellular free Ca^2+^ levels, which enables the Ca^2+^ to participate in various cellular processes, such as proliferation, metabolism and gene transcription. Ca^2+^ also has a multifunctional role in directional sensing, cytoskeleton redistribution, traction force generation and relocation of focal adhesions in tumor cells (19). However, the precise role of TRPV2 in bladder cancer remains unclear. Based on previous studies (20–22), we inferred that TRPV2 activity may be mediated by the direct regulation of key proteins, such as MMP2, which are used by cancer cells for invasion. MMP2 is associated with bladder cancer invasiveness (23) and its expression is often used to measure the migration ability of tumor cells. It was observed that MMP2 expression was significantly higher in 5637-TRPV2 cells than in the cells of the other two groups (Fig. 5). MMP2 is a Zn^2+^-dependent type IV collagenase with a molecular mass of 72 kDa. It is activated by biochemical interaction with a transmembrane MMP, called membrane-type (MT)-MMP, or by binding with integrin αVβl cell surface adhesion receptors. Numerous studies have demonstrated that MMP2 is critical in cancer development and progression (21,24–27). Cell migration is a complex process that requires the coordinated regulation of cell-cell attachment, cell-matrix attachment and matrix remodeling. MMP2 directly modulates cell-matrix adhesion by removing adhesion sites or by exposing binding sites to induce cell migration (28), and it affects tumor cell behavior *in vivo*, due to the ability to cleave growth factors, cell surface receptors, cell adhesion molecules and chemokines/cytokines, which promotes tumor metastases (29–31). Furthermore, MMP2 selects more aggressive phenotypes by generating apoptosis-resistant cells via the cleavage of proapoptotic factors (32), in addition to collaborating with other MMPs to promote cancer-related angiogenesis. As a result of these functions and roles, MMP2 is an extremely important protein in bladder cancer development and progression. The results of the present study suggest that MMP2 expression is increased during TRPV2 overexpression in 5637 cells, which is consistent with the previously described inference.

In conclusion, the nonselective cationic TRPV2 channel enhances bladder cancer cell migration, but does not affect cell proliferation *in vitro*. Furthermore, TRPV2 activity, which may be mediated by direct MMP2 regulation, is important in bladder tumor development and progression. These results suggest that TRPV2 channels are a potential target for therapeutic approaches to bladder carcinoma. However, the precise role of TRPV2 in bladder cancer *in vivo* requires further study.

## Figures and Tables

**Table I.** List of primers used for RT-PCR amplification.

Primer	5′-forward-3′	5′-reverse-3′	Product size (bp)	Accession no.
TRPV2 (a)	GTGACGGAACAGCCCACGGT	CAGTGATGCCTGGCCCTGATGG	475	NM_001270797.1
TRPV2 (b)	AACAAGGGGAAGCAGGAACCGC	GGCATTGACGAGGGGCTTGGG	390	NM_001270797.1
GAPDH	CGCTCCTGGAAGATGGTGAT	ACGGATTTGGTCGTATTGGG	214	NM_002046.4

RT-PCR, reverse transcription-polymerase chain reaction;TRPV2, transient receptor potential vanilloid 2; GAPDH, glyceraldehyde 3-phosphate dehydrogenase.

## Abbreviations:

TRP: transient receptor potential channel;
MMP2: matrix metalloproteinase 2
